# 10α-Hy­droxy-4,9-dimethyl-13-(morph­o­lin-4-ylmeth­yl)-3,8,15-trioxatetra­cyclo­[10.3.0.0^2,4^.0^7,9^]penta­decan-14-one

**DOI:** 10.1107/S1600536811053207

**Published:** 2011-12-17

**Authors:** Mohamed Moumou, Ahmed Benharref, Abdelghani Oudahmane, Fouad Mellouki, Moha Berraho

**Affiliations:** a bLaboratoire de Chimie Biomoléculaire, Substances Naturelles et Réactivité, URAC 16, Faculté des Sciences Semlalia, BP 2390, Bd My Abdellah, 40000 Marrakech, Morocco; b Universite Blaise Pascal, Laboratoire des Mate’riaux Inorganiques, UMR CNRS 6002, 24 Avenue des Landais, 63177 Aubie‘re, France; c Laboratoire de Chimie Bioorganique et Analytique, URAC 22, BP 146, FSTM, Université Hassan II, Mohammedia-Casablanca 20810 Mohammedia, Morocco

## Abstract

The title compound, C_19_H_29_NO_6_, was synthesized from 9α-hy­droxy­parthenolide (9α-hy­droxy-4,8-dimethyl-12-methyl­ene-3,14-dioxatricyclo­[9.3.0.0^2,4^]tetra­dec-7-en-13-one), which was isolated from the chloro­form extract of the aerial parts of *Anvillea radiata*. The mol­ecule contains a fused five- and ten-membered ring system. The ten-membered ring adopts an approximate chair–chair conformation, while the five-membered ring is in an envelope conformation, with the C atom closest to the hy­droxy group forming the flap. In the crystal, weak C—H⋯O hydrogen bonds connect the mol­ecules into layers parallel to (001). An intra­molecular O—H⋯N hydrogen bond is also present.

## Related literature

For background to the medicinal uses of the plant *Anvillea radiata*, see: El Hassany *et al.* (2004 ▶); Qureshi *et al.* (1990 ▶). For the reactivity of this sesquiterpene, see: Hwang *et al.* (2006 ▶); Neukirch *et al.* (2003 ▶); Neelakantan *et al.* (2009 ▶). For ring puckering parameters, see: Cremer & Pople (1975 ▶). For the synthesis see: Moumou *et al.* (2010 ▶).
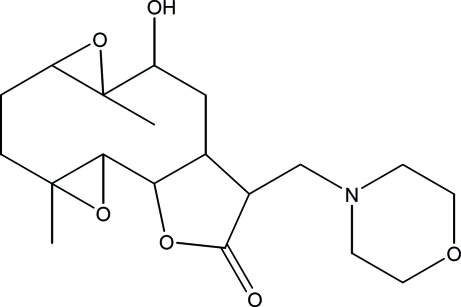


## Experimental

### 

#### Crystal data

C_19_H_29_NO_6_
*M* *_r_* = 367.43
Monoclinic, 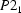

*a* = 11.6772 (9) Å
*b* = 6.9524 (4) Å
*c* = 11.8244 (9) Å
β = 102.160 (2)°
*V* = 938.42 (12) Å^3^
*Z* = 2
Mo *K*α radiation
μ = 0.10 mm^−1^
*T* = 296 K
0.65 × 0.45 × 0.26 mm

#### Data collection

Bruker X8 APEXII CCD diffractometer
7793 measured reflections
2069 independent reflections
1661 reflections with *I* > 2σ(*I*)
*R*_int_ = 0.032

#### Refinement

*R*[*F* ^2^ > 2σ(*F* ^2^)] = 0.044
*wR*(*F* ^2^) = 0.110
*S* = 1.07
2069 reflections
239 parameters
1 restraint
H-atom parameters constrained
Δρ_max_ = 0.20 e Å^−3^
Δρ_min_ = −0.23 e Å^−3^

### 

Data collection: *APEX2* (Bruker, 2005 ▶); cell refinement: *SAINT* (Bruker, 2005 ▶); data reduction: *SAINT*; program(s) used to solve structure: *SHELXS97* (Sheldrick,2008 ▶); program(s) used to refine structure: *SHELXL97* (Sheldrick,2008 ▶); molecular graphics: *ORTEP-3 for Windows* (Farrugia,1997 ▶)and *PLATON* (Spek, 2009 ▶); software used to prepare material for publication: *WinGX* (Farrugia, 1999 ▶).

## Supplementary Material

Crystal structure: contains datablock(s) I, global. DOI: 10.1107/S1600536811053207/lh5393sup1.cif

Structure factors: contains datablock(s) I. DOI: 10.1107/S1600536811053207/lh5393Isup2.hkl

Supplementary material file. DOI: 10.1107/S1600536811053207/lh5393Isup3.cml

Additional supplementary materials:  crystallographic information; 3D view; checkCIF report

## Figures and Tables

**Table 1:** Hydrogen-bond geometry (Å, °)

*D*—H⋯*A*	*D*—H	H⋯*A*	*D*⋯*A*	*D*—H⋯*A*
O1—H1*A*⋯N	0.82	2.23	3.048 (3)	178
C1—H1⋯O2^i^	0.98	2.32	3.169 (4)	145
C2—H2⋯O5^ii^	0.98	2.50	3.260 (3)	134
C4—H4*B*⋯O3^iii^	0.97	2.52	3.367 (4)	146

Symmetry codes: (i) 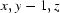; (ii) 
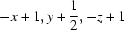
; (iii) 
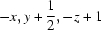
.

**Fig. 1.**
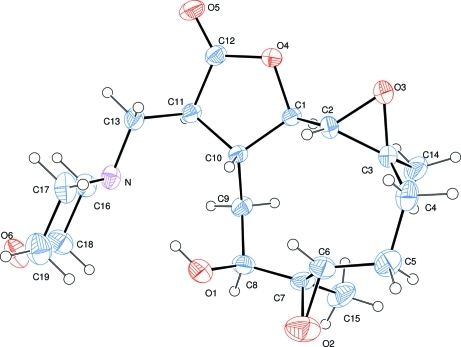
: Molecular structure of the title compound with displacement ellipsoids drawn at the 30% probability level. H atoms are represented as small spheres of arbitrary radii.

**Fig. 2.**
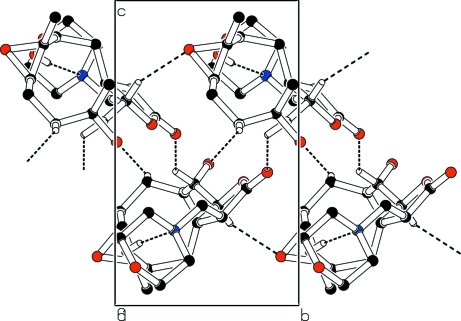
: Packing view showing the C–H···O and O–H···N hydrogen bonds as dashed lines. H atoms not involved in hydrogen bonding have been omitted for clarity.

## supplementary crystallographic information

### Comment

Our work lies within the framework of the valorization of medicinals plants and concerns *Anvillea radiata*. The main constituent of the chloroform extract of aerial parts of this plant is 9α - hydroxypartenolide (El Hassany *et al.*, 2004). The reactivity of this sesquiterpene lactone and its derivatives have been the subject of several studies (Neukirch *et al.*, 2003; Hwang *et al.*, 2006; Neelakantan *et al.*, 2009), in order to prepare products of value which can be used in the pharmacological industry. In this context, we have synthesed 9α-hydroxyparthenolide from 6β,7α-epoxy-9alpha hydoxy partenolide (9α-hydroxy-4,8-dimethyl-12- methylen-3,14-dioxa-tricyclo[9.3.0.0^2^,^4^] tetradec-7-en-13-one) (Moumou *et al.*, 2010) and then prepared the title compond (I). The crystal structure of (I) is determined herein. The molecule contains a fused ring system and morpholine group as a substituent to a lactone ring. The molecular structure (Fig.1) shows that the lactone ring adopts an envelope conformation, as indicated by Cremer & Pople (1975) puckering parameters Q = 0.189 (3)Å and φ = 66.0 (8)°. The ten-membered ring displays an approximate chair-chair conformation, while the morpholine ring has a perfect chair conformation with Q_T_ = 0.567 (4) Å, θ = 0.0 (3)° and φ2 = 157 (43)°. In the crystal structure, molecules are connected through weak C—H···O hydrogen bonds (Fig.2), forming layers parallel to (001). In addition, an intramolecular O—H···N hydrogen bond is also observed.

### Experimental

A mixture of 6β,7α-epoxy-9apha hydoxy partenolide (0.5 g, 1.89 mmol) and one equivalent of morpholine in EtOH (20 ml) was stirred for one twelve hours at room temperature. Then the reaction was stopped by adding water (10 ml) and extracted three times with ethyl acetate (3 *x* 20 ml). The combined organic layers were dried over anhydrous MgSO_4_, filtered and concentrated under vacuum to give 628 mg (1.79 mmol) of the title compound which was recrystallized from ethyl acetate.

### Refinement

All H atoms were fixed geometrically and treated as riding with C—H = 0.96 Å (methyl), 0.97 Å (methylene), 0. 98Å (methine) with *U*_iso_(H) = 1.2*U*_eq_ (methylene, methine) or *U*_iso_(H) = 1.5*U*_eq_ (methyl, OH). In the absence of significant anomalous scattering, the absolute configuration could not be reliably determined and thus the Friedel pairs were merged.

### Crystal data

**Table:**

C_19_H_29_NO_6_	*F*(000) = 396
*M**_r_* = 367.43	*D*_x_ = 1.300 Mg m^−^^3^
Monoclinic, *P*2_1_	Mo *K*α radiation, λ = 0.71073 Å
Hall symbol: P 2yb	Cell parameters from 2070 reflections
*a* = 11.6772 (9) Å	θ = 3.6–26.4°
*b* = 6.9524 (4) Å	µ = 0.10 mm^−^^1^
*c* = 11.8244 (9) Å	*T* = 296 K
β = 102.160 (2)°	Prism, colourless
*V* = 938.42 (12) Å^3^	0.65 × 0.45 × 0.26 mm
*Z* = 2	

### Data collection

**Table:**

Bruker X8 APEXII CCD diffractometer	1661 reflections with *I* > 2σ(*I*)
Radiation source: fine-focus sealed tube	*R*_int_ = 0.032
graphite	θ_max_ = 26.4°, θ_min_ = 3.6°
φ and ω scans	*h* = −14→14
7793 measured reflections	*k* = −6→8
2069 independent reflections	*l* = −14→13

### Refinement

**Table:**

Refinement on *F*^2^	Secondary atom site location: difference Fourier map
Least-squares matrix: full	Hydrogen site location: inferred from neighbouring sites
*R*[*F*^2^ > 2σ(*F*^2^)] = 0.044	H-atom parameters constrained
*wR*(*F*^2^) = 0.110	*w* = 1/[σ^2^(*F*_o_^2^) + (0.0604*P*)^2^ + 0.079*P*] where *P* = (*F*_o_^2^ + 2*F*_c_^2^)/3
*S* = 1.07	(Δ/σ)_max_ < 0.001
2069 reflections	Δρ_max_ = 0.20 e Å^−^^3^
239 parameters	Δρ_min_ = −0.23 e Å^−^^3^
1 restraint	Extinction correction: *SHELXL97* (Sheldrick,2008), Fc^*^=kFc[1+0.001xFc^2^λ^3^/sin(2θ)]^-1/4^
Primary atom site location: structure-invariant direct methods	Extinction coefficient: 0.047 (7)

### Special details

**Table:**

Geometry. All s.u.'s (except the s.u. in the dihedral angle between two l.s. planes) are estimated using the full covariance matrix. The cell s.u.'s are taken into account individually in the estimation of s.u.'s in distances, angles and torsion angles; correlations between s.u.'s in cell parameters are only used when they are defined by crystal symmetry. An approximate (isotropic) treatment of cell s.u.'s is used for estimating s.u.'s involving l.s. planes.
Refinement. Refinement of *F*^2^ against ALL reflections. The weighted *R*-factor *wR* and goodness of fit *S* are based on *F*^2^, conventional *R*-factors *R* are based on *F*, with *F* set to zero for negative *F*^2^. The threshold expression of *F*^2^ > 2σ(*F*^2^) is used only for calculating *R*-factors(gt) *etc*. and is not relevant to the choice of reflections for refinement. *R*-factors based on *F*^2^ are statistically about twice as large as those based on *F*, and *R*- factors based on ALL data will be even larger.

### Fractional atomic coordinates and isotropic or equivalent isotropic displacement parameters (Å^2^)

**Table:**

	*x*	*y*	*z*	*U*_iso_*/*U*_eq_	
C1	0.2787 (2)	0.2053 (4)	0.3279 (2)	0.0354 (6)	
H1	0.2200	0.1396	0.2692	0.042*	
C2	0.2207 (2)	0.3348 (5)	0.3984 (2)	0.0410 (7)	
H2	0.2724	0.4347	0.4397	0.049*	
C3	0.0963 (2)	0.3897 (5)	0.3702 (3)	0.0481 (8)	
C4	0.0687 (3)	0.5894 (6)	0.4046 (3)	0.0631 (10)	
H4A	0.1265	0.6277	0.4724	0.076*	
H4B	−0.0073	0.5888	0.4256	0.076*	
C5	0.0673 (3)	0.7374 (5)	0.3081 (4)	0.0659 (10)	
H5A	−0.0014	0.7161	0.2472	0.079*	
H5B	0.0615	0.8655	0.3389	0.079*	
C6	0.1748 (3)	0.7262 (4)	0.2578 (3)	0.0525 (8)	
H6	0.2486	0.7177	0.3153	0.063*	
C7	0.1814 (3)	0.6562 (4)	0.1444 (3)	0.0503 (8)	
C8	0.2969 (3)	0.5873 (5)	0.1194 (3)	0.0468 (7)	
H8	0.2923	0.6064	0.0365	0.056*	
C9	0.3194 (3)	0.3721 (5)	0.1433 (2)	0.0419 (7)	
H9A	0.3758	0.3287	0.0993	0.050*	
H9B	0.2469	0.3033	0.1145	0.050*	
C10	0.3649 (2)	0.3155 (4)	0.2708 (2)	0.0323 (6)	
H10	0.3866	0.4339	0.3151	0.039*	
C11	0.4738 (2)	0.1839 (4)	0.2903 (2)	0.0378 (6)	
H11	0.4747	0.1113	0.2195	0.045*	
C12	0.4563 (2)	0.0491 (4)	0.3832 (2)	0.0439 (7)	
C13	0.5891 (2)	0.2916 (5)	0.3277 (2)	0.0455 (7)	
H13A	0.5919	0.3489	0.4030	0.055*	
H13B	0.6533	0.2006	0.3355	0.055*	
C14	0.0086 (3)	0.2978 (6)	0.2748 (3)	0.0673 (11)	
H14A	0.0412	0.1824	0.2501	0.101*	
H14B	−0.0109	0.3854	0.2109	0.101*	
H14C	−0.0609	0.2666	0.3024	0.101*	
C15	0.0772 (3)	0.5911 (7)	0.0548 (3)	0.0753 (11)	
H15A	0.0068	0.6419	0.0731	0.113*	
H15B	0.0737	0.4531	0.0540	0.113*	
H15C	0.0845	0.6368	−0.0200	0.113*	
C16	0.6476 (3)	0.3626 (5)	0.1469 (3)	0.0481 (7)	
H16A	0.5893	0.2747	0.1048	0.058*	
H16B	0.7193	0.2908	0.1745	0.058*	
C17	0.6915 (3)	0.5830 (6)	0.3049 (3)	0.0568 (9)	
H17A	0.7644	0.5178	0.3374	0.068*	
H17B	0.6628	0.6422	0.3678	0.068*	
C18	0.6700 (3)	0.5208 (5)	0.0673 (3)	0.0610 (9)	
H18A	0.6983	0.4652	0.0031	0.073*	
H18B	0.5970	0.5870	0.0361	0.073*	
C19	0.7136 (3)	0.7371 (6)	0.2215 (3)	0.0697 (10)	
H19A	0.6419	0.8085	0.1934	0.084*	
H19B	0.7721	0.8263	0.2617	0.084*	
N	0.60588 (19)	0.4434 (4)	0.24620 (18)	0.0419 (6)	
O1	0.39115 (18)	0.7008 (3)	0.1779 (2)	0.0641 (7)	
H1A	0.4495	0.6332	0.1973	0.096*	
O2	0.1778 (3)	0.8609 (4)	0.1609 (3)	0.0845 (9)	
O3	0.14029 (17)	0.2566 (4)	0.46360 (19)	0.0613 (7)	
O4	0.34823 (15)	0.0656 (3)	0.40522 (16)	0.0440 (5)	
O5	0.5259 (2)	−0.0639 (4)	0.4365 (2)	0.0649 (7)	
O6	0.7532 (2)	0.6551 (4)	0.1254 (2)	0.0678 (7)	

### Atomic displacement parameters (Å^2^)

**Table:**

	*U*^11^	*U*^22^	*U*^33^	*U*^12^	*U*^13^	*U*^23^
C1	0.0331 (13)	0.0335 (14)	0.0376 (14)	0.0022 (11)	0.0032 (10)	0.0047 (12)
C2	0.0336 (13)	0.0482 (17)	0.0435 (16)	−0.0015 (12)	0.0135 (12)	0.0037 (14)
C3	0.0325 (14)	0.0540 (19)	0.0604 (19)	0.0025 (14)	0.0156 (13)	0.0134 (17)
C4	0.0520 (19)	0.073 (2)	0.072 (2)	0.0145 (18)	0.0297 (17)	−0.003 (2)
C5	0.070 (2)	0.0451 (19)	0.089 (3)	0.0156 (18)	0.0315 (19)	−0.004 (2)
C6	0.0608 (18)	0.0330 (17)	0.064 (2)	−0.0023 (14)	0.0126 (15)	−0.0011 (15)
C7	0.0543 (17)	0.0281 (15)	0.066 (2)	0.0054 (13)	0.0064 (15)	0.0079 (14)
C8	0.0523 (17)	0.0445 (17)	0.0430 (16)	0.0003 (14)	0.0084 (13)	0.0106 (14)
C9	0.0461 (15)	0.0467 (16)	0.0324 (14)	0.0087 (13)	0.0071 (12)	0.0003 (13)
C10	0.0349 (13)	0.0317 (14)	0.0306 (13)	0.0050 (11)	0.0074 (10)	0.0016 (11)
C11	0.0388 (14)	0.0434 (16)	0.0328 (14)	0.0095 (12)	0.0115 (11)	0.0020 (12)
C12	0.0424 (15)	0.0452 (17)	0.0443 (16)	0.0096 (14)	0.0098 (13)	0.0037 (14)
C13	0.0366 (14)	0.065 (2)	0.0364 (15)	0.0050 (14)	0.0110 (11)	0.0041 (15)
C14	0.0379 (16)	0.060 (2)	0.096 (3)	0.0009 (16)	−0.0017 (17)	0.015 (2)
C15	0.058 (2)	0.084 (3)	0.071 (2)	0.012 (2)	−0.0148 (17)	0.008 (2)
C16	0.0527 (16)	0.0531 (18)	0.0422 (16)	0.0025 (15)	0.0182 (13)	−0.0040 (15)
C17	0.0459 (16)	0.073 (2)	0.0537 (18)	−0.0100 (17)	0.0146 (14)	−0.0156 (19)
C18	0.071 (2)	0.063 (2)	0.0545 (19)	−0.0047 (18)	0.0258 (17)	0.0017 (18)
C19	0.066 (2)	0.062 (2)	0.083 (3)	−0.0115 (19)	0.0228 (19)	−0.017 (2)
N	0.0367 (12)	0.0561 (15)	0.0351 (12)	0.0013 (11)	0.0122 (10)	−0.0053 (12)
O1	0.0548 (13)	0.0511 (14)	0.0851 (17)	−0.0106 (11)	0.0121 (12)	0.0156 (13)
O2	0.114 (2)	0.0458 (15)	0.095 (2)	0.0084 (15)	0.0268 (18)	0.0058 (15)
O3	0.0436 (11)	0.0799 (17)	0.0678 (14)	0.0123 (11)	0.0285 (10)	0.0279 (14)
O4	0.0425 (11)	0.0441 (11)	0.0470 (11)	0.0054 (9)	0.0135 (9)	0.0138 (9)
O5	0.0584 (13)	0.0708 (17)	0.0663 (14)	0.0282 (13)	0.0148 (11)	0.0282 (13)
O6	0.0679 (14)	0.0695 (16)	0.0750 (15)	−0.0158 (13)	0.0351 (12)	−0.0037 (14)

### Geometric parameters (Å, °)

**Table:**

C1—O4	1.458 (3)	C11—C12	1.490 (4)
C1—C2	1.484 (4)	C11—C13	1.523 (4)
C1—C10	1.530 (3)	C11—H11	0.9800
C1—H1	0.9800	C12—O5	1.208 (3)
C2—O3	1.441 (3)	C12—O4	1.346 (3)
C2—C3	1.471 (4)	C13—N	1.470 (4)
C2—H2	0.9800	C13—H13A	0.9700
C3—O3	1.449 (4)	C13—H13B	0.9700
C3—C14	1.498 (5)	C14—H14A	0.9600
C3—C4	1.500 (5)	C14—H14B	0.9600
C4—C5	1.535 (5)	C14—H14C	0.9600
C4—H4A	0.9700	C15—H15A	0.9600
C4—H4B	0.9700	C15—H15B	0.9600
C5—C6	1.500 (5)	C15—H15C	0.9600
C5—H5A	0.9700	C16—N	1.474 (4)
C5—H5B	0.9700	C16—C18	1.506 (5)
C6—C7	1.443 (5)	C16—H16A	0.9700
C6—O2	1.486 (4)	C16—H16B	0.9700
C6—H6	0.9800	C17—N	1.460 (4)
C7—O2	1.438 (4)	C17—C19	1.514 (5)
C7—C15	1.505 (5)	C17—H17A	0.9700
C7—C8	1.518 (4)	C17—H17B	0.9700
C8—O1	1.411 (4)	C18—O6	1.417 (4)
C8—C9	1.535 (4)	C18—H18A	0.9700
C8—H8	0.9800	C18—H18B	0.9700
C9—C10	1.540 (4)	C19—O6	1.432 (4)
C9—H9A	0.9700	C19—H19A	0.9700
C9—H9B	0.9700	C19—H19B	0.9700
C10—C11	1.544 (3)	O1—H1A	0.8200
C10—H10	0.9800		
			
O4—C1—C2	108.2 (2)	C12—C11—C13	110.2 (2)
O4—C1—C10	106.18 (18)	C12—C11—C10	104.2 (2)
C2—C1—C10	111.4 (2)	C13—C11—C10	113.7 (2)
O4—C1—H1	110.3	C12—C11—H11	109.5
C2—C1—H1	110.3	C13—C11—H11	109.5
C10—C1—H1	110.3	C10—C11—H11	109.5
O3—C2—C3	59.70 (17)	O5—C12—O4	120.6 (3)
O3—C2—C1	119.8 (3)	O5—C12—C11	127.7 (3)
C3—C2—C1	125.2 (3)	O4—C12—C11	111.6 (2)
O3—C2—H2	113.8	N—C13—C11	113.2 (2)
C3—C2—H2	113.8	N—C13—H13A	108.9
C1—C2—H2	113.8	C11—C13—H13A	108.9
O3—C3—C2	59.13 (17)	N—C13—H13B	108.9
O3—C3—C14	112.2 (3)	C11—C13—H13B	108.9
C2—C3—C14	123.1 (3)	H13A—C13—H13B	107.7
O3—C3—C4	116.5 (3)	C3—C14—H14A	109.5
C2—C3—C4	115.9 (3)	C3—C14—H14B	109.5
C14—C3—C4	116.5 (3)	H14A—C14—H14B	109.5
C3—C4—C5	112.8 (3)	C3—C14—H14C	109.5
C3—C4—H4A	109.0	H14A—C14—H14C	109.5
C5—C4—H4A	109.0	H14B—C14—H14C	109.5
C3—C4—H4B	109.0	C7—C15—H15A	109.5
C5—C4—H4B	109.0	C7—C15—H15B	109.5
H4A—C4—H4B	107.8	H15A—C15—H15B	109.5
C6—C5—C4	112.5 (3)	C7—C15—H15C	109.5
C6—C5—H5A	109.1	H15A—C15—H15C	109.5
C4—C5—H5A	109.1	H15B—C15—H15C	109.5
C6—C5—H5B	109.1	N—C16—C18	110.4 (3)
C4—C5—H5B	109.1	N—C16—H16A	109.6
H5A—C5—H5B	107.8	C18—C16—H16A	109.6
C7—C6—O2	58.8 (2)	N—C16—H16B	109.6
C7—C6—C5	126.8 (3)	C18—C16—H16B	109.6
O2—C6—C5	115.5 (3)	H16A—C16—H16B	108.1
C7—C6—H6	114.4	N—C17—C19	110.8 (3)
O2—C6—H6	114.4	N—C17—H17A	109.5
C5—C6—H6	114.4	C19—C17—H17A	109.5
O2—C7—C6	62.1 (2)	N—C17—H17B	109.5
O2—C7—C15	110.5 (3)	C19—C17—H17B	109.5
C6—C7—C15	124.2 (3)	H17A—C17—H17B	108.1
O2—C7—C8	113.0 (3)	O6—C18—C16	111.7 (3)
C6—C7—C8	120.7 (3)	O6—C18—H18A	109.3
C15—C7—C8	112.8 (3)	C16—C18—H18A	109.3
O1—C8—C7	111.2 (3)	O6—C18—H18B	109.3
O1—C8—C9	111.7 (2)	C16—C18—H18B	109.3
C7—C8—C9	113.3 (3)	H18A—C18—H18B	107.9
O1—C8—H8	106.7	O6—C19—C17	111.3 (3)
C7—C8—H8	106.7	O6—C19—H19A	109.4
C9—C8—H8	106.7	C17—C19—H19A	109.4
C8—C9—C10	116.1 (2)	O6—C19—H19B	109.4
C8—C9—H9A	108.3	C17—C19—H19B	109.4
C10—C9—H9A	108.3	H19A—C19—H19B	108.0
C8—C9—H9B	108.3	C17—N—C13	109.8 (2)
C10—C9—H9B	108.3	C17—N—C16	108.9 (2)
H9A—C9—H9B	107.4	C13—N—C16	111.1 (2)
C1—C10—C9	115.9 (2)	C8—O1—H1A	109.5
C1—C10—C11	103.6 (2)	C7—O2—C6	59.1 (2)
C9—C10—C11	113.6 (2)	C2—O3—C3	61.17 (18)
C1—C10—H10	107.8	C12—O4—C1	110.9 (2)
C9—C10—H10	107.8	C18—O6—C19	110.1 (2)
C11—C10—H10	107.8		

### Hydrogen-bond geometry (Å, °)

**Table:**

*D*—H···*A*	*D*—H	H···*A*	*D*···*A*	*D*—H···*A*
O1—H1A···N	0.82	2.23	3.048 (3)	178
C1—H1···O2^i^	0.98	2.32	3.169 (4)	145
C2—H2···O5^ii^	0.98	2.50	3.260 (3)	134
C4—H4B···O3^iii^	0.97	2.52	3.367 (4)	146

Symmetry codes:  (i) *x*, *y*−1, *z*; (ii) −*x*+1, *y*+1/2, −*z*+1; (iii) −*x*, *y*+1/2, −*z*+1.
